# A Handheld Quantifiable Soft Tissue Manipulation Device for Tracking Real-Time Dispersive Force-Motion Patterns to Characterize Manual Therapy Treatment

**DOI:** 10.1109/TBME.2022.3222124

**Published:** 2023-04-20

**Authors:** Abhinaba Bhattacharjee, Sohel Anwar, Stanley Chien, M. Terry Loghmani

**Affiliations:** Department of Mechanical and Energy Engineering, Purdue School of Engineering and Technology, USA.; Department of Mechanical and Energy Engineering, Purdue School of Engineering and Technology, USA.; Department of Electrical and Computer Engineering, Purdue School of Engineering and Technology, USA.; Physical Therapy Department, Indiana University School of Health and Human Sciences, Indianapolis, IN 46202 USA

**Keywords:** Neuromusculoskeletal disorders, soft tissue manipulation, medical device, force-motion patterns, treatment parameters, massage, physical therapy, precision rehabilitation

## Abstract

**Objective:**

Low back pain (LBP) is one of the leading neuromusculoskeletal (NMSK) problems around the globe. Soft Tissue Manipulation (STM) is a force-based, non-invasive intervention used to clinically address NMSK pain conditions. Current STM practice standards are mostly subjective, suggesting an urgent need for quantitative metrics. This research aims at developing a handheld, portable smart medical device for tracking real-time dispersive force-motions to characterize manual therapy treatments as Quantifiable Soft Tissue Manipulation (QSTM).

**Methods:**

The device includes two 3D load-cells to quantify compressive and planar-shear forces, coupled with a 6 degrees-of-freedom IMU sensor for acquiring volitionally adapted therapeutic motions while scanning and mobilizing myofascial restrictions over larger areas of the body. These force-motions characterize QSTM with treatment parameters (targeted force, application angle, rate, direction, motion pattern, time) as a part of post-processing on a PC software (Q-Ware©). A human case study was conducted to treat LBP as proof-of-concept for the device’s clinical usability.

**Results:**

External validation of treatment parameters reported adequate device precision required for clinical use. The case study findings revealed identifiable therapeutic force-motion patterns within treatments indicating subject’s elevated force-endurance with self-reported pain reduction.

**Conclusion:**

QSTM metrics may enable study of STM dosing for optimized pain reduction and functional outcomes using documentable manual therapy. Clinical trials will further determine its reliability and comparison to conventional STM.

**Significance:**

This medical device technology not only advances the state-of-the-art manual therapy with precision rehabilitation but also augments practice with reproducibility to examine neurobiological responses of individualized STM prescriptions for NMSK pathology.

## I. Introduction

NEUROMUSCULOSKELETAL (NMSK) problems escalate alarmingly with aging, producing chronic pain, joint dysfunction or immobility [1]. These chronic NMSK conditions may lead to major surgeries with complicating medications and expensive healthcare visits. Therefore, it is essential to advance non-pharmacological and non-invasive alternatives to traditional medical approaches. Developing non-addictive, force-based therapeutic modalities that enable quantitative measures to address pain is a high priority for the National Institutes of Health (NIH). Soft Tissue Manipulation (STM) [2], [3], a non-invasive force-based intervention, constitutes an integral part of manual therapy which facilitates treatment of NMSK conditions. It enables a therapist to manually palpate and locate soft tissue restrictions or scar tissues and treat them with externally applied forces in linear or curvilinear fashion, shown to remediate inflammation [4] and enhance blood flow and vascularity. With the current state-of-the-art STM practice, palpation and treatment are performed either by hand only or using tools made of steel or wood for Instrument-Assisted Soft Tissue Manipulation (IASTM).

The penetrable capacity of contoured tooltips, i.e., treatment edges of IASTM tools [5] can offer resonance-based reverberations to a clinician’s hands. This magnifies soft tissue palpation extensively for detecting underlying tissue structures and irregularities. Consistent IASTM on a rodent model proved to enhance healing efficiencies [4], [6] of soft tissue injuries. Human studies [7], [8], [9] with IASTM over a stipulated time also revealed positive implications on the biomechanical properties and neurological behavior of soft tissues. But much remains to be understood about the underlying mechanisms related to clinical treatment parameters [10] that are needed to achieve optimal outcomes.

Current research often uses robotic/mechatronic laboratory setups for mimetic-massage [11], [12] applied on small animals in a uniaxial direction, at targeted area of interest, revealing positive biological outcomes. Additionally, some human studies have applied targeted STM forces [13], [14]. However, these methods are either not portable, maneuverable, or durable enough to capture the complex STM force-motions as practically performed by clinicians over multiple areas and body regions. Maintaining targeted pressure consistency along with the motion pattern progression at a reliable pace are fundamental components needed to advance the art of conventional STM. Furthermore, its importance in facilitating students’ ability to reproduce an instructor’s technique during training is apparent. The lack of scientific rigor to objectively measure STM makes practice reliant mainly on subjective patient-therapist feedback and interactions during treatment. This unrecorded STM is neither adequately documentable nor sufficiently replicable for future reference. This deficiency may devalue the full potential of manual therapy and suggests the urgent need for its characterization with objective, quantitative metrics during realistic STM applications in support of individualized, precision rehabilitation. Quantitative measures are required to better document, monitor, adjust, and progress soft tissue intervention, enable consistent targeted force, capture angular orientation of force application, treatment rate and force-motion pattern progressions for reproducibility in between treatment sessions and users (e.g., clinicians, researchers, instructor-students), and effectively compare results. Therefore, addition of real-time sensory tactile motion feedback to IASTM tools mitigates this deficiency and conceives Quantifiable Soft Tissue Manipulation (QSTM).

Tactile sensing [15], [16] is common in palpating probes of robot-assisted minimally invasive surgery [17], [18] used for tumor localization [18] or stiffness mapping [19]. Nevertheless, these probes aren’t designed for adaptive maneuverable therapeutic STM force applicability over wider areas of interest required for treating clinical NMSK conditions. The rate of change of angular force delivery on soft tissue layers over timed intervals using several force-motion signatures in varying paces of application, form the basis of our research and emphasizes the need to quantify STM objectively. Integrating digital technology with IASTM, we introduce a novel portable handheld smart medical device for evaluating dynamic adaptive continuous real-time dispersive force-motions of manual therapy using Quantifiable Soft Tissue Manipulation (QSTM).

Related work includes a handheld device by Everingham et al. [20] for examining uniaxial mechanical loading parameters to investigate effects of targeted compressive force feedback and stroke frequencies on tissue healing in a rodent model. It lacks triaxial force sensing paradigms with no device pose estimation and the setup is too cumbersome for clinical practice. A precursor to findings presented in this paper was studied by Alotaibi et al. [21] for determining triaxial forces and pose orientations for basic characterization of IASTM application, while studying its stress/strain analysis on a computer simulated human tissue model [22]. Moreover, Thistle et al. [23] examined dispersive IASTM device handle’s pressure distribution for analysis of clinician discomforts and hand fatigues of specific handholds over prolonged treatment sessions.

Our prior work incorporates development of a localized force-motion sensing QSTM medical device [24], with a half-disc shaped tapered tooltip specialized for treating smaller regions of interests (digits, wrists, foot/ankle, myofascial trigger points/painful foci, etc.) with shorter massage stroke lengths. This localized device in conjunction with a dispersive QSTM device, operated by a customized clinical PC software (Q-Ware©) [24], [25], constitutes a comprehensive manual therapy device system needed for patient care. This paper is an extension to our prior work [24], [25], focusing on the handheld dispersive QSTM device equipped with an elongated convex treatment blade for dynamic force-motion applications as sustained during lengthy stroking treatments over wider and broader surface areas of the body. This purpose of this paper is to present the dispersive handheld device’s system architecture, working methodology with 3D force and orientation tracking, force-motion gravity correction, and their characterization into QSTM treatment parameters for treatment documentation and replication. The clinical usability of this device system is validated and changes in the soft tissue quality and clinical outcomes discussed in an institutional review board approved case study on a human subject with chronic low back pain for proof-of concept. Furthermore, the STM-dose regimen is elaborated in support of the clinical efficacy of using this technology for assessment and treatment of NMSK pain disorders.

## II. System Architecture

The dispersive handheld QSTM device is designed to scan wider areas of body (e.g., shoulder, upper and lower back, thighs, and lower extremity), for locating soft tissue lesions or fascial restrictions. Adaptive targeted force-motions using dispersive QSTM device eventually helps release or mobilize such soft tissue abnormalities, promoting blood flow, and attaining painless mobility.

### A. Hardware Design

**Fig. 1.**
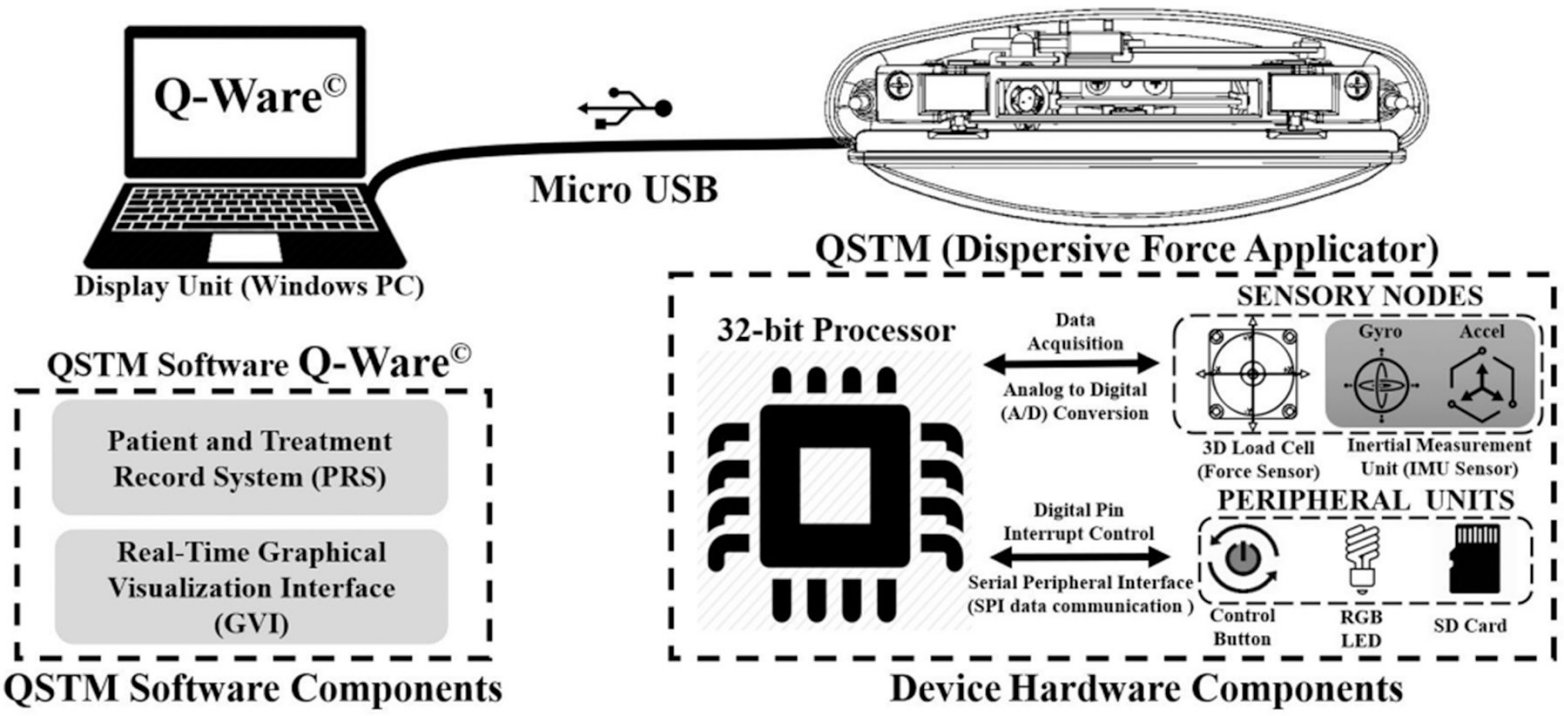
System architecture of handheld dispersive QSTM device elaborated with all hardware and software components.

Illustrations on the treatment blade, the ergonomics of the handheld part, and the positioning and connectivity of sensors & peripheral electronics constituting the device are mentioned here. A long, stainless steel convex treatment blade, with a 30° beveled edge is designed to enable dispersive force application with large, sweeping, or arched force-motions for scanning broader areas and palpating intricate soft tissue restrictions. The ergonomic long and narrow handle of the device provides a solid double handed grip to the user, with the USB data cable emerging from the middle at the rear side of the handle. The handle also includes a flange-like extension on both the front and back side of the external casing to prevent unwanted touching of the blade during treatment. This feature helps to avoid force measurement skewedness due to finger interference on the blade. The blade is connected to two 3D load cells (Tech Gihan USL-AP) through two load shafts, such that the force exerted at the blade is distributed on the six channels of both 3D load cells. The load cells are secured inside a rigid encapsulation, to provide structural integrity and stabilized force sensing. This encapsulation along with a 9DOF MARG sensor (ICM-20948 TDK InvenSense Inc.) as Inertial Measuring Unit (IMU), a 32bit ARMv7 NXP processor and additional peripheral electronics (Control button, SD card and RGB LED) are wired together and compactly packaged inside the casing. The device uses USB serial communication to transmit force and motion data to a Personal Computer PC, as shown in Fig. 1. The weight of the convex blade is approximately 250 grams. As the blade is suspended on the load-shafts, the device must rest on a calibration cradle with its convex edge facing downwards for calibration before usage. The physical alignment of both the load cell axes and the IMU sensor axes was challenging and took several iterations to be finalized for pose-based gravity compensation using force-motion transformations.

### B. Software Design

**Fig. 2.**
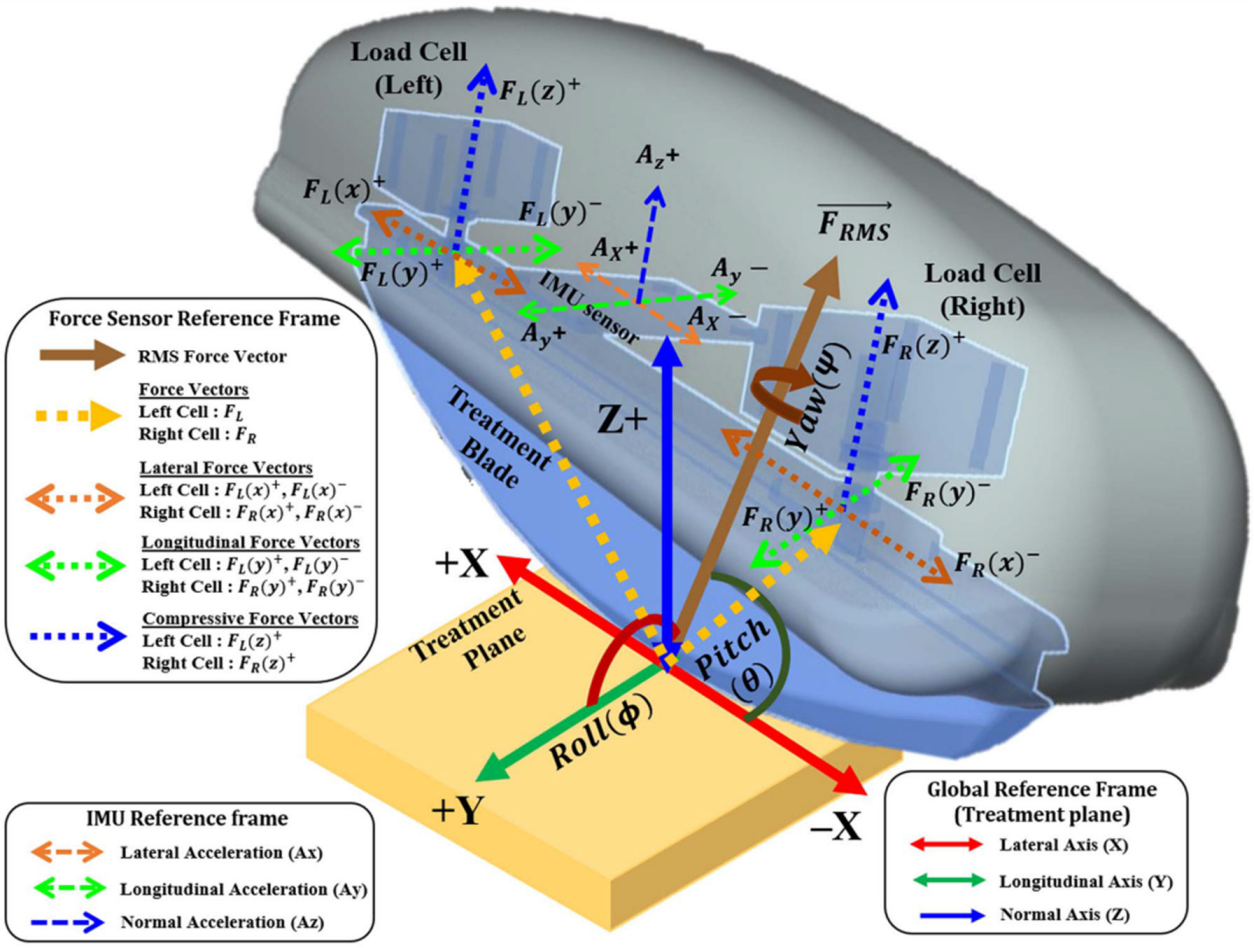
Positioning of the sensors (3D load cells and IMU sensor) inside the dispersive handheld device which represents the local sensor reference frame with respect to the global reference frame. The 2D treatment plane is horizontally aligned to the global reference frame.

The computations of the QSTM medical device system are distributed between an Embedded Firmware on a microcontroller in the device and a PC Software (Q-Ware©) developed to operate multiple QSTM medical devices for clinical use. The embedded firmware is a multithreaded application. It performs sensor-data acquisition, device calibration, force quantification, device tilt sensing, and serial communication to PC. Additionally, the firmware executes control button-based interrupt service routines for switching operational states during the treatment mode of the device. The six axes’ measurements from two 3D load cells are transformed into three force components i.e., compressive (vertical-Z) normal to the treatment plane and planar (shear-X and tensile-Y) along the lateral and longitudinal direction of the blade’s point of contact. As shown in Fig. 2, the average magnitude of the RMS Force vector forms the resultant Dose-Load of every force-motion stroke cycle applied during treatment. Whereas Q-Ware©, operating on PC, being a multi-processing software executes multiple tasks. It features a local Patient-Treatment Record System (PRS) for patient treatment data management; a device-specific Graphical Visual Interface (GVI) for real-time 3D force and motion tracking and data monitoring; and a Data Analysis Interface for visual comparison or assessment of several force-motion portfolios applied during treatment. In addition, a Treatment Retrieval System for importing recorded data is incorporated for referencing and assessment of patient progress.

## III. Methodology

**Fig. 3.**
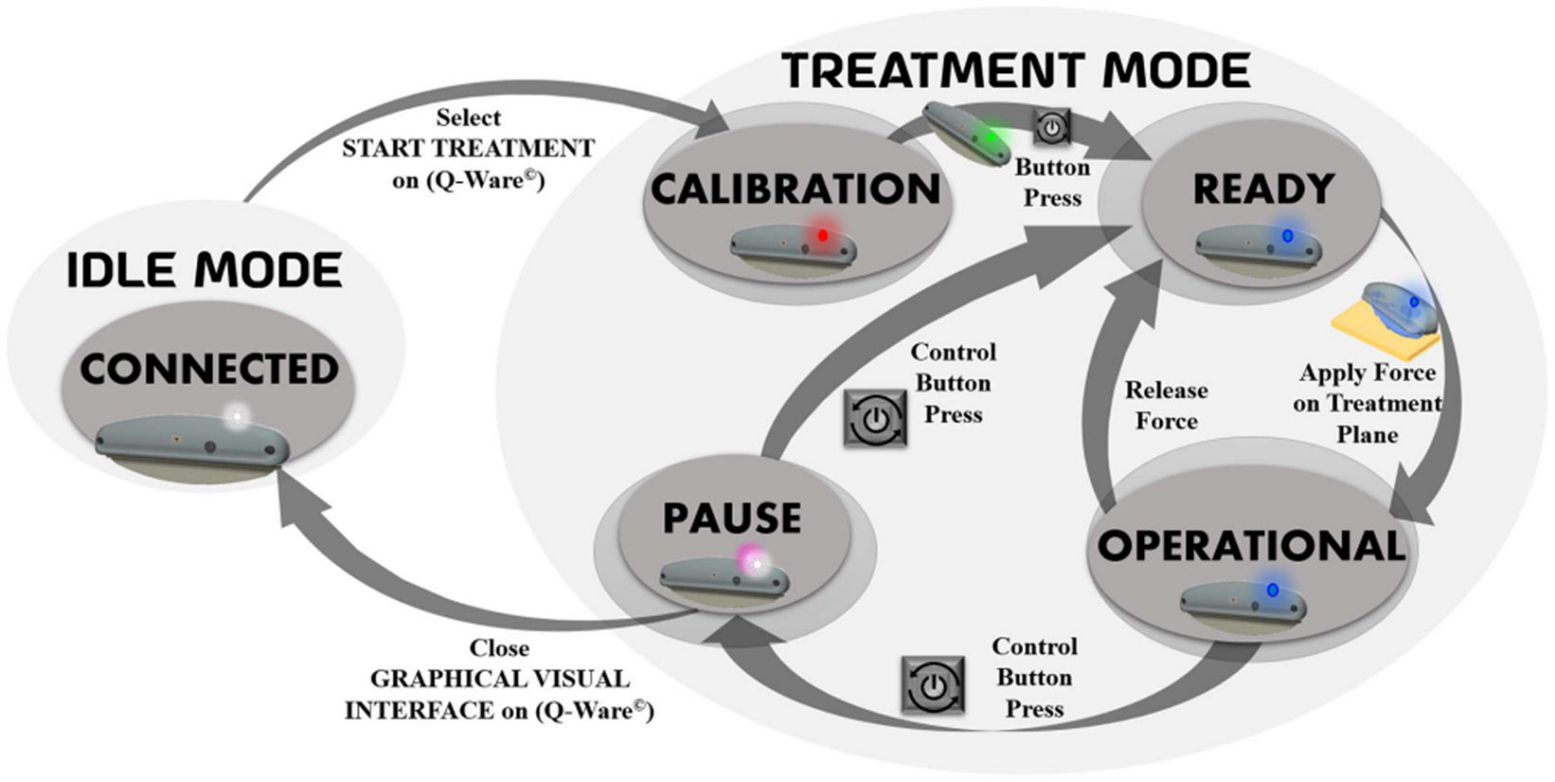
Diagrammatic illustration of handheld device’s operation with system modes and states.

**Fig. 4.**
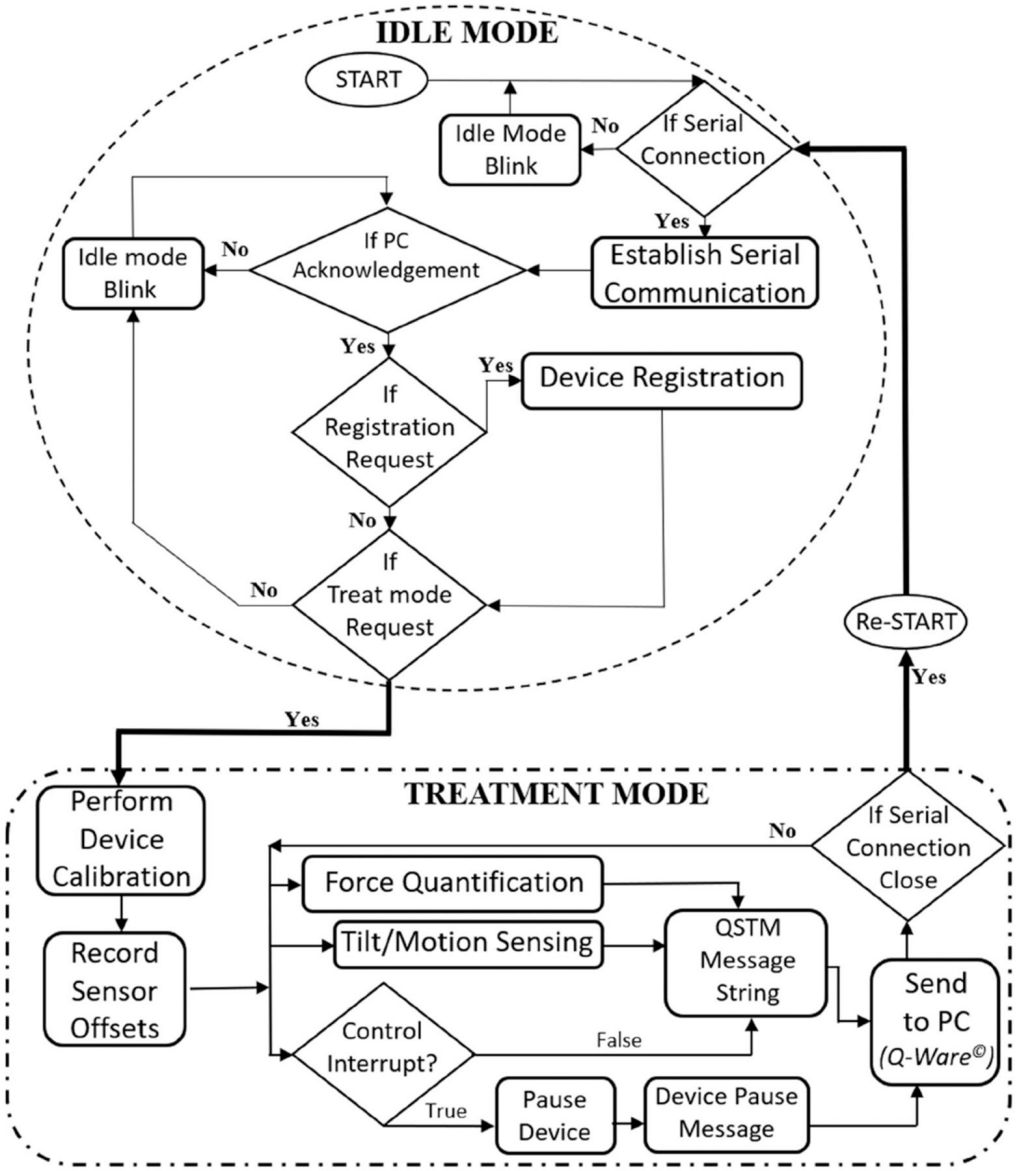
Diagrammatic illustration of the workflow of embedded firmware elaborating the methodology of system operation modes.

The handheld device operates in two system modes (Idle mode and Treatment Mode), as shown in Fig. 3. The Treatment mode has four states, i.e., calibration, ready, operational and pause states. Fig. 4 explains the workflow of the tasks performed by the handheld device in its system modes. In Idle mode, the device first establishes serial communication with PC, registers itself on PC’s Q-Ware© with identification information, and then waits for user requests to start treatment mode. This mode is indicated by a 1 Hz white LED blink on the device, while resting on its cradle in a predefined position.

The Treatment mode starts with user’s “start treatment” selection on Q-Ware©, with a solid red LED glow, indicating the calibration state. The device should be left untouched during calibration, until a solid green LED glow indicates calibration is complete. After calibration, a control button press on the device starts the ready state. The treatment mode performs a multithreaded operation of three tasks – Tilt sensing with respect to gravity, 3D Force Quantification and executing Interrupt Service Routines (ISR) based on control button input. A solid blue LED glow indicates both the ready and operational state of treatment mode, while the device pause state, triggered by a device button press, is indicated by alternate white and pink LED blinks every second.

### A. Tilt Orientation Sensing With Respect to Gravity

The instantaneous device orientation angle is essential for determining the force application angle with respect to the gravity/global frame of reference. The 16bit 3D accelerometer and 3D gyroscope data, acquired from the IMU sensor are transmitted through 1^2^C communication protocol to the microprocessor. The 3D acceleration and 3D gyro biases are eliminated using bias offsets determined, in the calibration step. These 6 DOF data are fused at 200 Hz sampling rate to estimate precise orientation angles.

A conventional complementary filter-based approach [26] was initially implemented to find optimal location for positioning IMU sensor on the device. Observations from this approach led us to place the IMU sensor exactly at the center of the handheld device and align it with the 3D load cell lateral axis, as shown in Fig. 2 to avoid orientation mismatch. Eventually it is updated with quaternion transformations [27], [28] using the modified gradient descent-based Attitude Heading Reference System filter [29]. Solving the gimbal lock situation [30] and minimizing computation overhead are the major advantages of using quaternions over Euler angle-based computation. The filter uses a unit gain parameter (β=1) and is updated at a frequency of 200 Hz. The filter output yields a four-element unit quaternion vector explained in Eq. (1-2).

(1) $$Q_{o}+Q_{1}i+Q_{2}j+Q_{3}k$$

where $Q_{o}$ is the scalar quantity describing the rotation angle and $Q_{1}$, $Q_{2}$ & $Q_{3}$ are coefficients of axis-vector components describing the orientation in Euclidean space (i,j,k). So essentially, if a unit vector axis of rotation [x,y,z], is rotated by an angle α, then the quaternion for this rotation will be of the form:

(2) $$\cos\left(\frac{\alpha }{2}\right)+\sin\left(\frac{\alpha }{2}\right)(xi+yj+zk)$$

and the norm of all four components will be equal to 1. The elements of the unit quaternion are further transformed into Euler angles in degrees of the form Yaw (ψ), Pitch (θ) and Roll (∅); which are rotations about the Z, Y and X axes of the handheld device in global coordinate frame respectively. Eq. (3-5) explains the quaternion to Euler angle conversions to estimate real-time orientation angles of the device [27], [28].


(3) $$\psi =\tan^{-1}\left(\frac{2(Q_{1}Q_{2}+Q_{0}Q_{3})}{Q_{0}^{2}+Q_{1}^{2}-Q_{2}^{2}-Q_{3}^{2}}\right)\times \frac{180^{{}_{{}^{\circ}}}}{\pi }$$

(4) $$\theta =\sin^{-1}(-2(Q_{1}Q_{3}-Q_{0}Q_{2}))\times \frac{180^{{}_{{}^{\circ}}}}{\pi }$$

(5) $$\emptyset =\tan^{-1}\left(\frac{2(Q_{0}Q_{1}+Q_{2}Q_{3})}{Q_{0}^{2}-Q_{1}^{2}-Q_{2}^{2}+Q_{3}^{2}}\right)\times \frac{180^{{}_{{}^{\circ}}}}{\pi }$$

The output of this filter provides a fast response with almost no visualized lag of angular orientations minimizing latency and jitter. The gain parameter β is tuned to match the gyro bias for integration drift compensation and improve steady and random dynamic motion sensing accuracy.

### B. 3D Force Quantification

This section describes the voltage signal acquisition of 3D forces and conditioning in (6), and the transformation of the acquired voltage signal to force units (Newton) in (7)-(9) using the calculated offsets and calibration parameters. Finally, the obtained resultant force is further corrected to minimize the gravity effect of blade’s weight by kinematic transformations in (12)-(15) using rotation sequence in (10)-(11).

The analog output from 3D load cells, sampled at 500 Hz with 16bit resolution are recorded for offset voltage determination at the calibration step. The force offset voltage vector ($\vec{V_{off}}$) for both load cells are obtained by the measured mean of each of the six channels’ thermal noise distribution calculated during calibration (no-load condition of device). The difference of load-voltage vector $(\vec{V}{)}_{j}$, with dimension [x,y,z], from offset voltage vector $\vec{V_{off}}$ for each load cell, at the $j^{th}$ iteration, is fed into a rolling mean filter of sample size (n=25) for removing high-frequency channel noise.


(6) $$\vec{V_{F}}=\frac{1}{n}\sum_{j=0}^{n-1}{\left(\vec{V}\right)}_{j}-\vec{V_{off}}$$

The matrix representation of force-signal vector $\vec{V_{F}}$ from Eq. (6) is transformed into an actual force vector $\vec{F}$ in Newtons, by multiplying with (voltage-force) characterization matrix $A_{3}\times_{3}$, given by the load-cell manufacturer. The blade, being suspended from sensors exerts a tension force due to its weight along gravity direction. This causes an offset voltage baseline shift of ξ volts along the $F_{z}$ axis, aligned to gravity, at calibration. As a result, the device suffers force measurement skewness at other orthogonal axes when it is rotated in different orientations. The orthogonal force measurement skewness is resolved using a voltage correction, by subtracting a offset voltage calibration error (ξ volts) along gravity aligned to sensor axis at calibration orientation. This correction, shown in Eq. 7, ensures uniform orthogonal distribution of blade’s weight along the device’s local coordinate system.


(7) $$\left[\begin{array}{c} F_{x}\\ F_{y}\\ F_{z} \end{array}\right]=\left[\begin{array}{ccc} A_{11} & A_{12} & A_{13}\\ A_{21} & A_{22} & A_{23}\\ A_{31} & A_{32} & A_{33} \end{array}\right]\times \left[\begin{array}{c} V_{F(x)}\\ V_{F(y)}\\ V_{F(z)} \end{array}\right]\;\;-\left[\begin{array}{ccc} 1 & 0 & 0\\ 0 & 1 & 0\\ 0 & 0 & A_{33} \end{array}\right]\times \left[\begin{array}{c} 0\\ 0\\ \xi \end{array}\right]$$

Eq. (7) is deployed for voltage-force characterization and orthogonal force corrections of both left and right load cells. The individual force components of the respective left and right load cells are added up to yield a 3D force vector as shown in (8).


(8) $$\left[\begin{array}{c} F_{3D(x)}\\ F_{3D(y)}\\ F_{3D(z)} \end{array}\right]={\left[\begin{array}{c} F_{x}\\ F_{y}\\ F_{z} \end{array}\right]}_{L}+{\left[\begin{array}{c} F_{x}\\ F_{y}\\ F_{z} \end{array}\right]}_{R}$$

The 3D force components in Eq. (8) are root mean squared to achieve the resultant force of the handheld device.


(9) $$F_{RMS}=\sqrt{(F_{3D(x)}{)}^{2}+(F_{3D(Y)}{)}^{2}+(F_{3D(Z)}{)}^{2}}$$

Frms produces the resultant instantaneous force along the moving force co-ordinate system $F_{B}$ of the handheld device, where B represents the local reference frame of the device. The orthogonally distributed weight of blade adds tension forces along gravity direction when the device is rotated without applying forces. These tension forces present in the moving force coordinate system $F_{B}$ needs gravity correction. This correction is facilitated by the transformation of measured 3D forces from local device co-ordinate frame B into the global inertial co-ordinate frame I, by forward kinematic equations. Here, $F_{I}$ denotes the inertial force co-ordinate system, where all relative accelerations are assumed to be zero. A 3D rotation transformation matrix $R_{\left(\psi ,\theta ,\emptyset \right)}^{3\times 3}$, is derived using the combination of rotation angles from Eq. (3), Eq. (4) & Eq. (5) with Euler rotation combination sequence of ZYX axes:

(10) $$R_{\left(\psi ,\theta ,\emptyset \right)}^{3\times 3}=Rot_{Z}\;(\psi )\times Rot_{Y}(\theta {)}^{T}\times Rot_{X}\;(\emptyset )$$

(11) $$R_{\left(\psi ,\theta ,\emptyset \right)}^{3\times 3}\;=\left[\begin{array}{ccc} C_{\psi }C_{\theta } & C_{\psi }S_{\theta }S_{\emptyset }-S_{\psi }C_{\theta } & C_{\psi }S_{\theta }C_{\emptyset }+S_{\psi }S_{\emptyset }\\ C_{\psi }C_{\theta } & C_{\psi }C_{\emptyset }-S_{\psi }S_{\theta }S_{\emptyset } & -C_{\psi }S_{\emptyset }-S_{\psi }S_{\theta }C_{\emptyset }\\ S_{\theta } & C_{\theta }S_{\emptyset } & C_{\theta }C_{\emptyset } \end{array}\right]$$

where $Rot_{Z}(\psi )$, $Rot_{Y}(\theta )$, and $Rot_{X}(\emptyset )$ are the rotations about Z, Y & X axes, while $C_{\psi }=Cos(\psi )$ and $S_{\psi }=Sin(\psi )$ and corresponding sines and cosines of the other rotation angles. Now applying forward kinematics, the 3D force vector from Eq. (8) is converted from local to the inertial co-ordinate system using 3D transformation matrix from Eq. (11).

(12) $$F_{I(x,y,z)}^{B(3\times 1)}=R_{\left(\psi ,\theta ,\emptyset \right)}^{3\times 3}\times [F_{3D(x)}\;\;F_{3D(y)}\;\;F_{3D(z)}{]}^{T}$$

where $F_{I}^{B}$, represents the force transformation from local frame B to inertial frame I. Since the blade’s absolute weight ($M_{b}$ Newtons) is aligned along the $F_{Z}$ direction during initial calibration, it needs to be subtracted from the inertial frame’s Z component of the Force vector to eliminate the additive forces due to blade’s weight.


(13) $$F_{I(x,y,z{)}_{updated}}^{B(3\times 1)}=F_{I(x,y,z)}^{B(3\times 1)}-[0\;\;0\;\;M_{b}{]}^{T}$$

Finally, the updated inertial forces are transformed back to the moving force co-ordinate system of local reference frame by applying inverse kinematic transformation of the rotation matrix from Eq. (11).


(14) $$F_{B(x,y,z{)}_{updated}}^{I(3\times 1)}=[R_{\left(\psi ,\theta ,\emptyset \right)}^{3\times 3}{]}^{-1}\times F_{I(x,y,z{)}_{\mathrm{updated}}}^{B(3\times 1)}$$

The updated forces yield error diminished weight corrected measurements in all orientations, where the force noise levels at each axis are confined to 0.2 Newtons.


(15) $$F_{\;B(RMS)}^{I}=\sqrt{{\left(F_{B(x)}^{I}\right)}^{2}+{\left(F_{B(y)}^{I}\right)}^{2}+{\left(F_{B(z)}^{I}\right)}^{2}}$$

Hence the resultant force $F_{B(RMS)}^{I}$ forms the instantaneous dose-load of STM at every force-motion stroke cycle during a clinical treatment session.

### C. Determination of Device Contact With Skin

The magnitude of the resultant force $F_{B(RMS)}^{I}$ is harnessed to find a threshold for determining whether the device is in contact with skin or not. The treatment blade weighs 2.5N (~250 grams). Jerks or swift rotations might trigger sudden force due to inertial momentum. Henceforth the threshold magnitude is set to 1 Newton (much greater than the force noise level). Resultant forces above the threshold determine the operational state of treatment mode, while that below threshold indicates a ready state (device waiting to be used). The control button is used to switch the device from the Operational state to Pause state during treatment mode by an alternate button press. The Pause state is marked by an alternate pink and white LED blink. The sum of the time accounted for both Ready and Pause states of the device defines the dead time of the entire session.

### D. QSTM Treatment Parameters

The QSTM message string comprises of the 3D Force Vector $[F_{B(x)}^{I},F_{B(y)}^{I},F_{B(z)}^{I}{]}^{T}$, the Resultant Force $F_{B(RMS)}^{I}$, the geoorientation angles yaw (ψ), pitch(θ) and roll(∅) with respect to gravity, acceleration & gyro vectors from IMU, along with the control button state (High/Low). This string is sent to PC’s Q-Ware© at a serial transmission frequency of 100 Hz with a USB baud rate of 115.200 kbps. The quantified force-motions data delivered to Q-Ware© is processed to yield QSTM Treatment parameters. These parameters include average compressive force, average resultant force, maximum peak force (maximum of all local maxima in the resultant force stream), target force (average of all peak forces of all force-motion cycles during a treatment session matched with a user defined target), number of treatment strokes, skin-contact time, elapsed treatment time and stroke frequency.

### E. Treatment Stroke Detection and Rate Estimation

The Q-Ware© streams and displays the force and motion data on its Graphical Visualization Interface (GVI) for real-time visualization using a time-division multiplexing algorithm at a variable framerate. It also saves the raw data stream in a csv file for post-processing, future referencing, and analysis. The resultant force $F_{RMS}$ stream is first subjected to a sliding window Low Pass Filter (LPF), and then searched for local maxima and minima to generate force peak-valley pairs.

#### 1) Noise Filtering:

A digital low pass filter with a discreet binomial kernel, derived from the binomial distribution, of the form:

(16) $$F_{RMS(LPF)}=\sum_{k=0}^{n}\left(\left(\begin{array}{c} n\\ k \end{array}\right)\times \frac{\sqrt{n}}{2^{n}}\times F_{RMS}\right)∕\;\;\sum_{k=0}^{n}\left(\left(\begin{array}{c} n\\ k \end{array}\right)\times \frac{\sqrt{n}}{2^{n}}\right)$$

has been implemented to smooth noise frequencies of the original signal; where n is window size and k is the window iterator. Another higher order (N = 10) Butterworth filter using a cut-off frequency of 11 Hz was implemented to match the results of Binomial Kernel based LPF with window size (n = 25). The latter performs better for steady motions as compared to the former which achieves better signal to noise ratio for nondeterministic sporadic motions. Hence, there is a tradeoff in smoothing out noise due to hand vibrations during force application and retaining essential signal ripples observed due to tissue irregularities (tight spots, nodules) of the underlying skin contour.

#### 2) Treatment Stroke Determination:

For convenience, each Treatment Stroke is determined by the maximum resultant peak force per force-motion cycle, discarding the redundant peaks (due to hand vibrations/tissue irregularity) from stroke count consideration for every force-motion portfolio. Therefore, a decision tree-based algorithm is designed to eliminate redundant peaks and detect maximum force peak per cycle for stroke identification and treatment rate estimation.

#### 3) Decision Tree Based Treatment Rate Estimation:

**Fig. 5.**
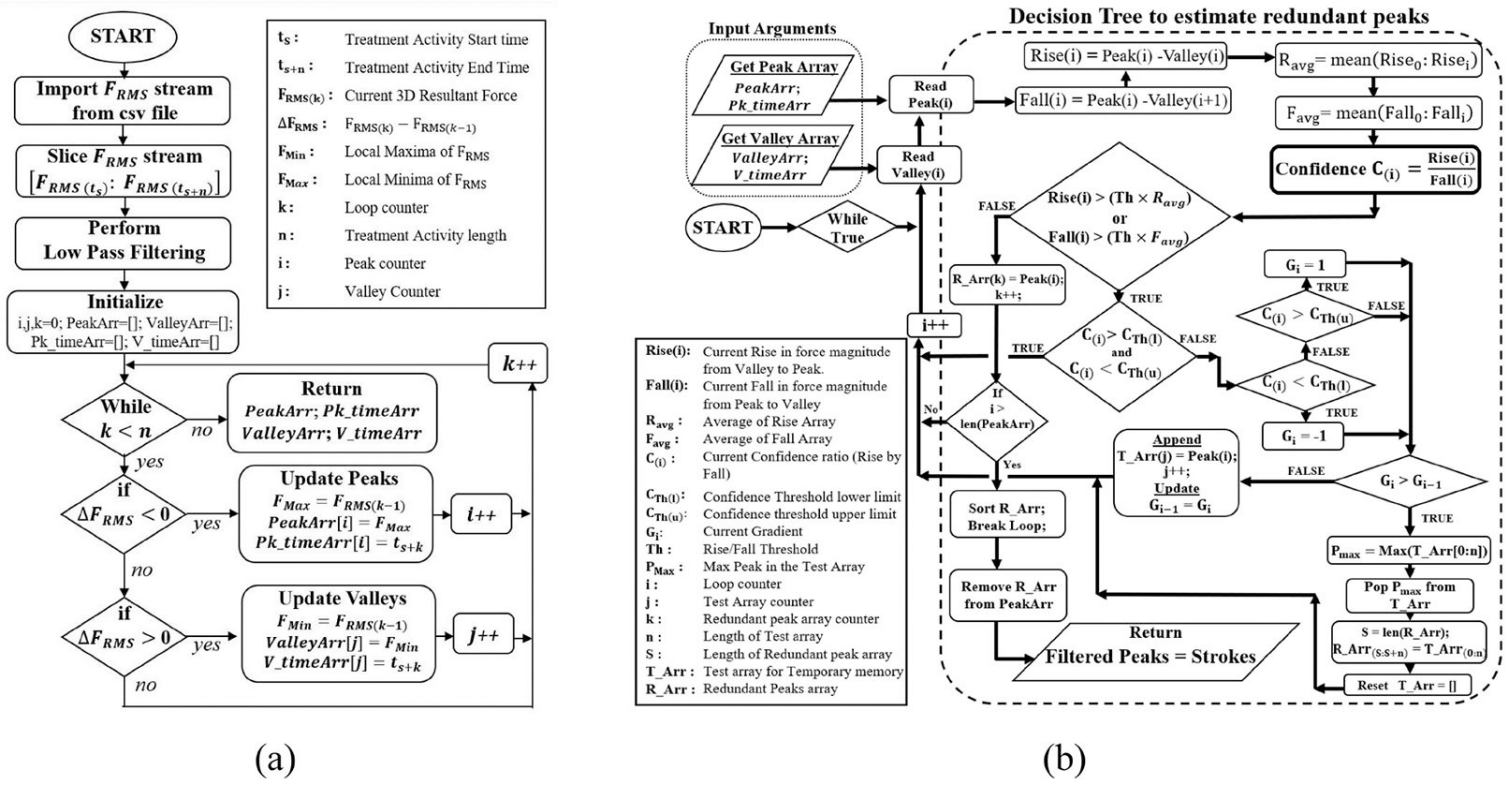
Flow charts representations for treatment stroke detection and rate estimation: (a) Flowchart identifying local maxima (Force Peak) & local minima (Force-Valley) from gradients of $F_{\text{RMS}}$ data stream. (b) Flowchart showing Decision tree-based algorithm to eliminate redundant force peaks per force-motion cycle due to hand vibrations or underlying irregularities in skin surface. This ensures each filtered peak as a stroke peak of that force-motion cycle and the stroke counts are summed up throughout the active time sequences to estimate the treatment rate of the session.

**Fig. 6.**
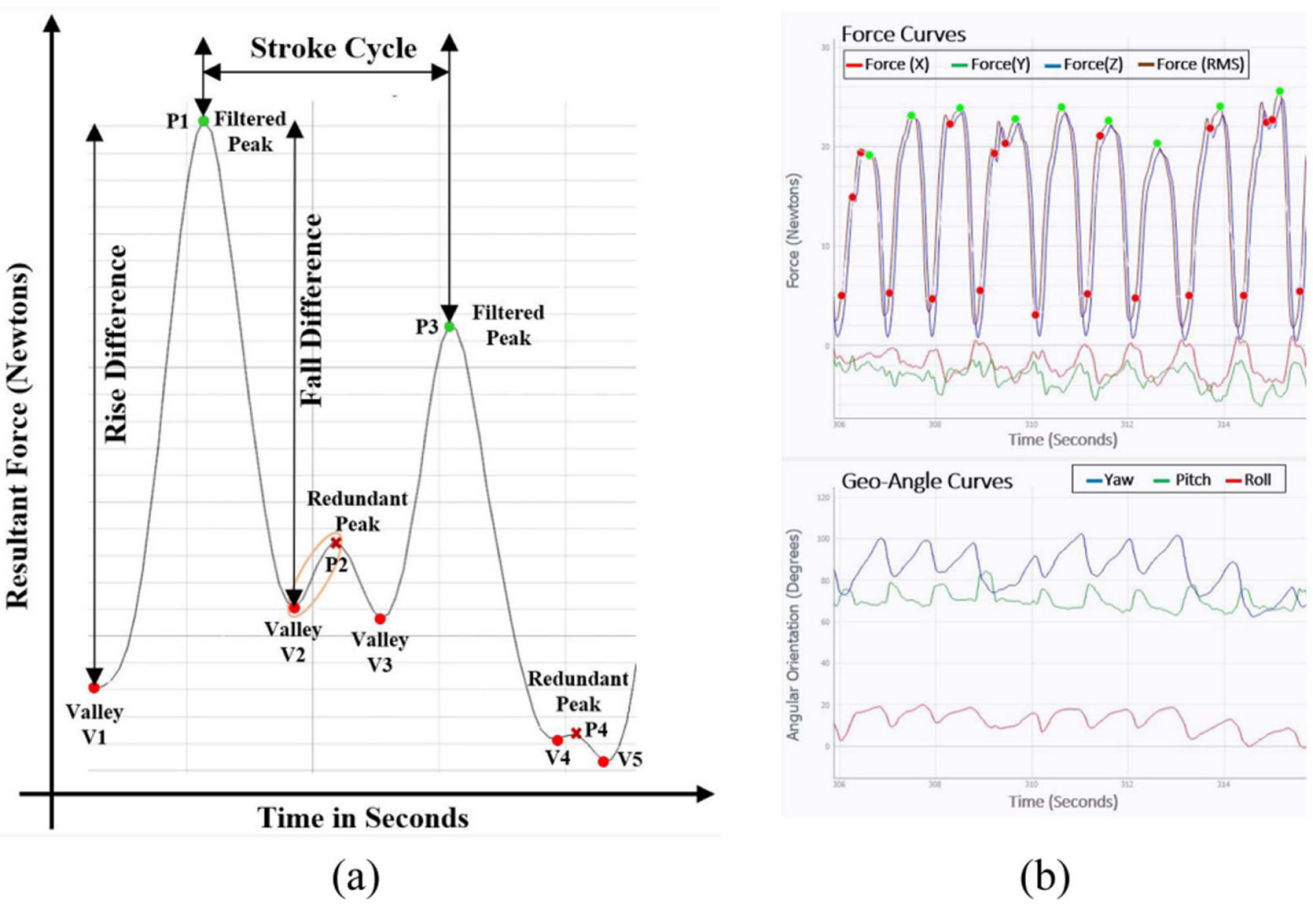
Force and motion waveforms. (a) Sliced resultant force waveform, indicating peak-valley pairs and distinguishing primary stroke peaks and redundant peaks. (b) Dynamic waveforms of time dependent 3D forces with corresponding 3D angular orientations depicting a force-motion progression.

The peak-valley pairs are generated from the gradients of the real-time filtered force data stream using the algorithm described in flowchart of Fig. 5(a). These peak-valley pairs form the fundamental features for the decision tree algorithm as shown in flowchart of Fig. 5(b). It computes a confidence ratio (ratio of rise in force magnitude from valley-peak to fall in force magnitude from peak-next valley) for each valley to peak to next valley ($V_{i}\text{−}P_{i}\text{−}V_{i+1}$) combinations. These confidence ratios for each combination are further thresholded with a range of confidence thresholds (determined experimentally based on graphical observations of force waveform patterns) to discard redundant peaks, shown in Fig. 6(a), thus conserving the primary peak force constituting every stroke cycle. The output of the algorithm yields the number of filtered peaks as number of strokes for the total contact time. A limitation of this technology in capturing the treatment motion-path traversed by the device with respect to the human body as an external reference is realized. Currently, the angular motion of the device is visualized by the change in yaw-pitch-roll data with respect to the stroke cycle. The stroke cycle is variable and depends on the volitional adaptations of motions of the user based on treatment. Fig. 6(b) represents the 3D force curve and 3D angular orientation (Geo-Angle) curve with respect to time in the form of waveforms taken from a treatment window. Distinct repetitions of change in 3D angular orientations of the device are evident from Geo-Angle curve in Fig. 6(b). Such repetitions form similar motion patterns performed by the therapist during a treatment.

The summation of strokes over a sequence of contact times for each force-motion portfolio is then calculated and divided by the total treatment time to yield the Treatment Stroke frequency which indicates the treatment rate. This information, along with the target force and the average treatment angle, is critical for determining the treatment type for personalized STM treatments.

## IV. Experiments and Results

**Fig. 7.**
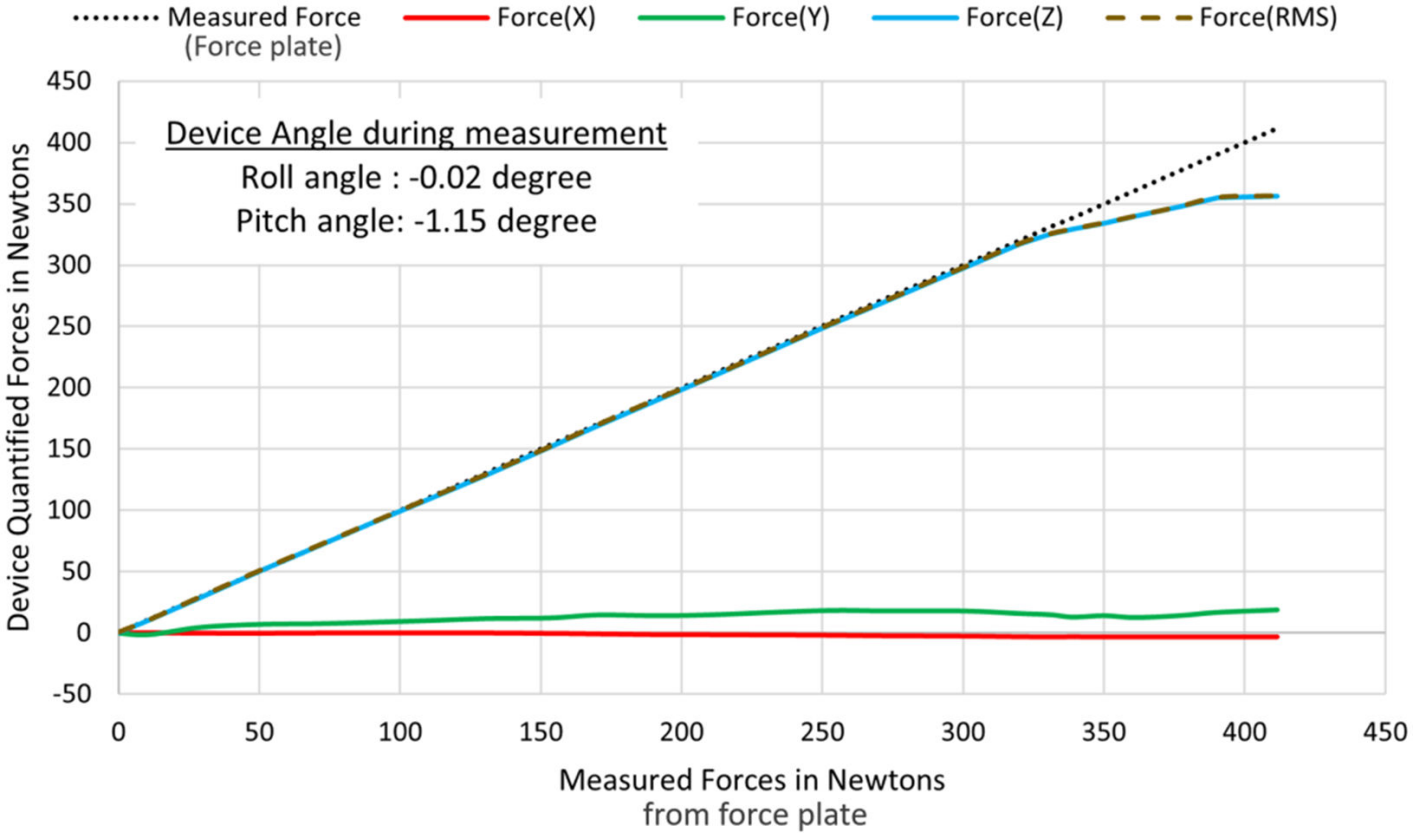
Force validation of compressive forces (predominant along Z direction) on force plate, indicating linearity and saturating at 325N – 360N. range.

**Fig. 8.**
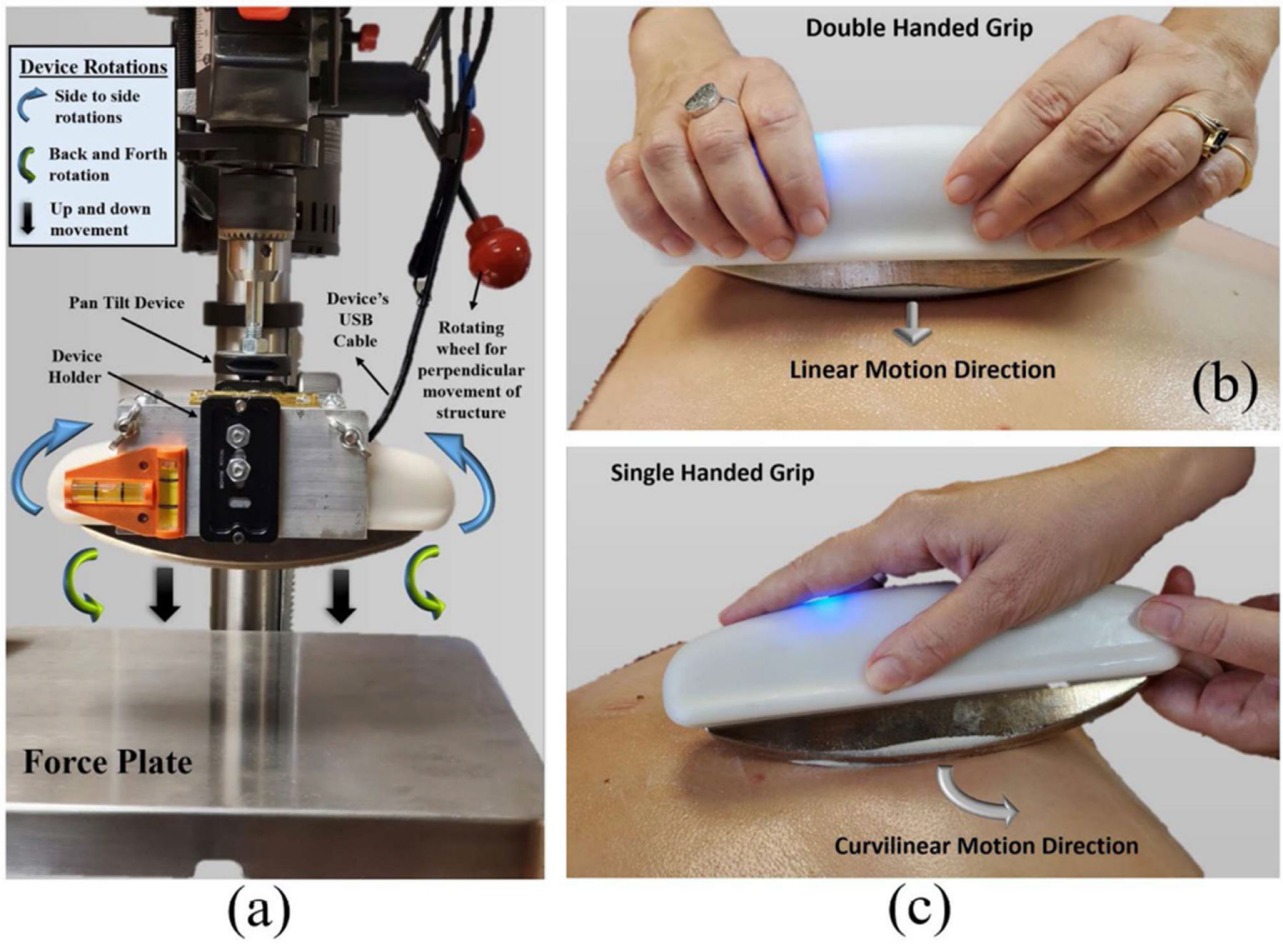
Clinical testing & force validation of handheld device on external force plate: (a) Test Rig with pan-tilt set up for validation of forces and angular orientations. (b) Double handed grip of the handheld device for performing linear motions. (c) Single handed grip of handheld device with second hand as acting as a pivot to perform curvilinear motions.

Two different versions of dispersive handheld devices were built with maximum 200N and 400N force measurement capacities, out of which the former saturates within 160N-180N compressive force range, while the latter measures up to 325N-360N range as shown in Fig. 7. The device’s estimated 3D rotation angles were validated by placing it on a manually operated pan-tilt calibration test rig as shown in Fig. 8(a). Several experiments have been performed to validate measured forces applied with different handgrips, especially, double handed grip and single-handed handhold as shown in Fig. 8(b) and (c). Moreover, measured steady forces were validated on an external force plate (PCE-PB-150N), of 0.5N measuring resolution, placed on the base of the test rig.

### A. Hardware Testing

**Fig. 9.**
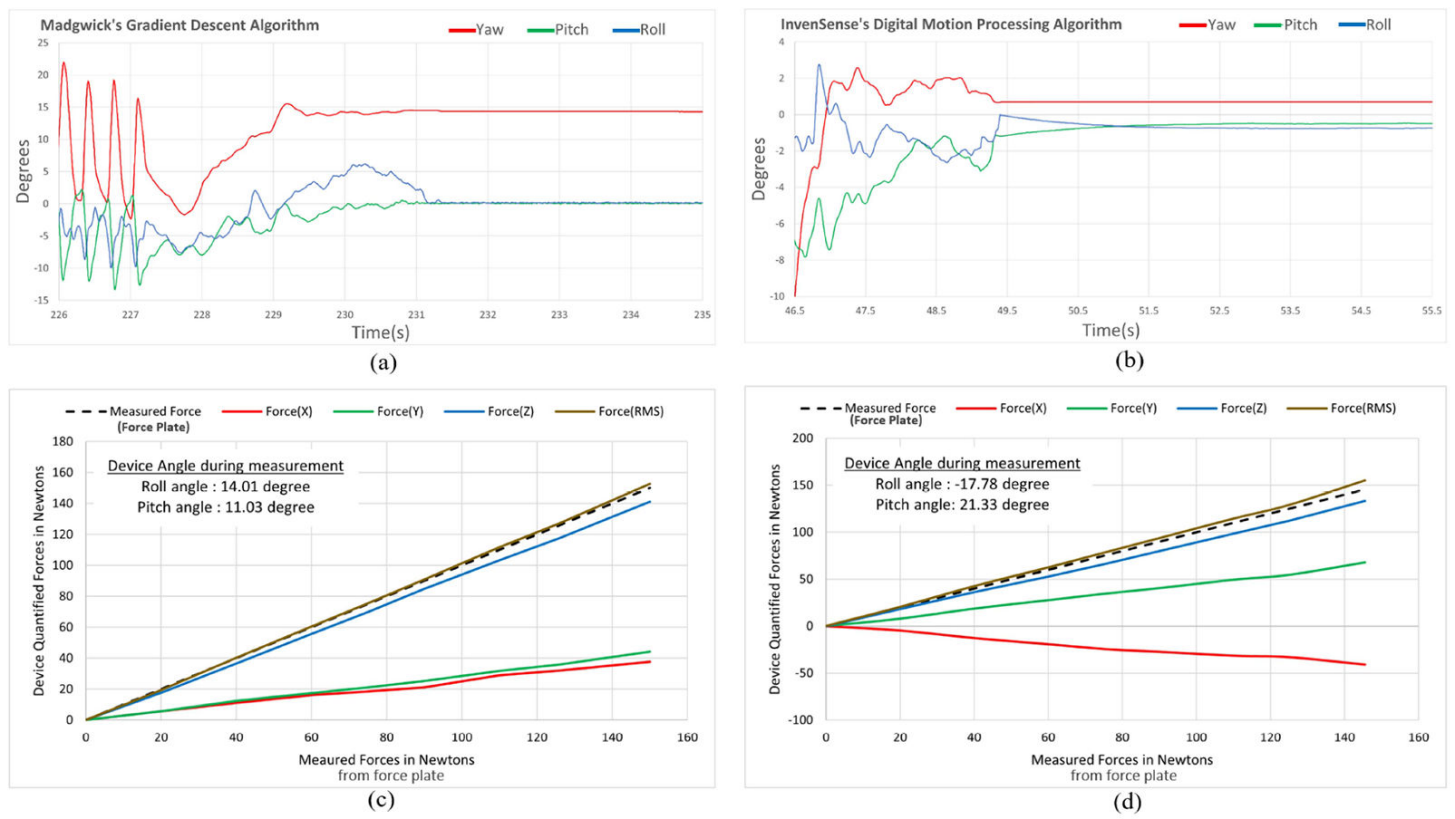
Comparisons of orientation algorithms with respect to dynamic non-deterministic motions and graphical representations showing linearity in force measurements at different angular orientations: (a) Drift of Yaw orientation angles calculated by Gradient Descent Algorithm, which doesn’t converge to zero at initial orientation position after suffering vibrational motion. (b) Improvements in orientation convergence by measurements from Digital Motion Processing Algorithm when device returns to initial position irrespective of vibrational motions. (c) Force validation at a right inclined orientation, where $F_{\text{RMS}}$ is compared with the measured forces in dashed line. (d) Force validation at left inclined orientation, where 3D forces are compared to the measured force in dashed line.

The load cells operate on 3.3V DC power, and the 16-bit analog to digital converter quantizes the measured voltages in approximately 0.02–0.03 mV range. This translates to device’s compressive (along Z axis) force resolution to be ~0.1N to 0.2N range based on the manufacturer’s calibration matrix ($A_{3\times 3}$), while that of planar (along X & Y axes) forces account to be about ±0.05N to ±0.1N range. The static and dynamic responses of Euler angle rotations were further validated on the Orientation Viewer of MATLAB’s Sensor Fusion Toolbox [31] and compared with its built-in Kalman filter based AHRS algorithms. The response of the Gradient Descent based orientation estimation AHRS filter proved to be effective for steady motions within 0–5 Hz range with a ±2.15% error range. Repeated observations of sporadic nondeterministic dynamic motion gestures (with jerks and flickers ~ > 5 Hz) produces a rotational drift more than ±10% error in measured Yaw angles, which adds up over prolonged usage. Implementation of InvenSense’s Digital Motion Processing (DMP) algorithm [32], comparison shown in Fig. 9(a) & 9(b), effectively compensates for this drift error reducing the error range to ±2%. Additional techniques for absolute pose estimation can be achieved by fusing 3D (North, East, Down) components from Magnetometer data into MARG filter [33] or Extended Complimentary AHRS filter [34], after calibrating for hard and soft iron offsets [35], [36] introduced due to environmental electromagnetic interferences. The linearity of device’s computed forces with respect to the measured forces on external force plate is evident from the graphs shown in Fig. 9(c) and 9(d). However, these graphs also reveal an approximate linearity in force measurement error with rising force magnitude measured at both orientations of the device. The error escalation can be minimized by observing the force response of the device mounted on a Robotic Arm, at different orientations and tuning the calibration parameters for optimal performance.

### B. Software Testing

**Fig. 10.**
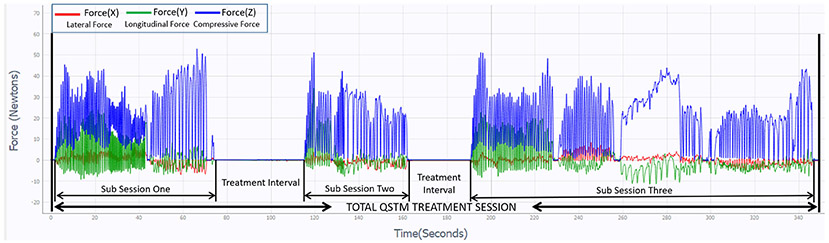
Graphical depiction of 3D force waveforms representing six minutes of treatment session with handheld dispersive QSTM device. The figure also indicates the discreet treatment sub-sessions (operational state), and treatment interval periods (pause state) in between sub-sessions during Treatment mode.

In prior work, experiments with the handheld dispersive device were performed on both inanimate padded surfaces and in rodents [4], [25]. The Institutional Review Board of Indiana University under protocol number 1408895969 approved human subjects clinical trials on 6^th^ August, 2021 for assessing the clinical impact of QSTM (in progress). Visual observations from the graphical 3D force-time waveforms as shown in Fig. 10 represents the operational state i.e., treatment sub-sessions, and the pause state (treatment interval in between sub-sessions) during a six-minute treatment session on a human subject. Different force-motion stroke patterns combining planar (longitudinal and lateral), and compressive forces, collectively constitutes the STM Dose-load regimen for consistent and variable frequencies over stipulated contact times, as administered by the clinician. The magnitude and frequency of these compressive and planar forces synchronously (in phase) or asynchronously (out of phase) applied on the skin directly impacts the underlying soft tissue properties. Hence, the average resultant force magnitude of every force-motion cycle and combinations of their 3D components defines a unit Dose-load per motion pattern. The force-motion patterns are of important clinical significance, as cells and tissues are highly sensitive to different external stimuli (compressive, tensile or shear stresses). The train of these consecutive similar paced force-motion stroke patterns within a sub-session are called treatment bursts in clinical terms. A series of treatment bursts constitute a treatment sub-session (operational state of device i.e., skin contact time between two pause states). The sequence of these treatment sub-sessions (operational state) and the interposing treatment interval (pause state) add up to the total treatment time called the treatment session.

**Fig. 11.**
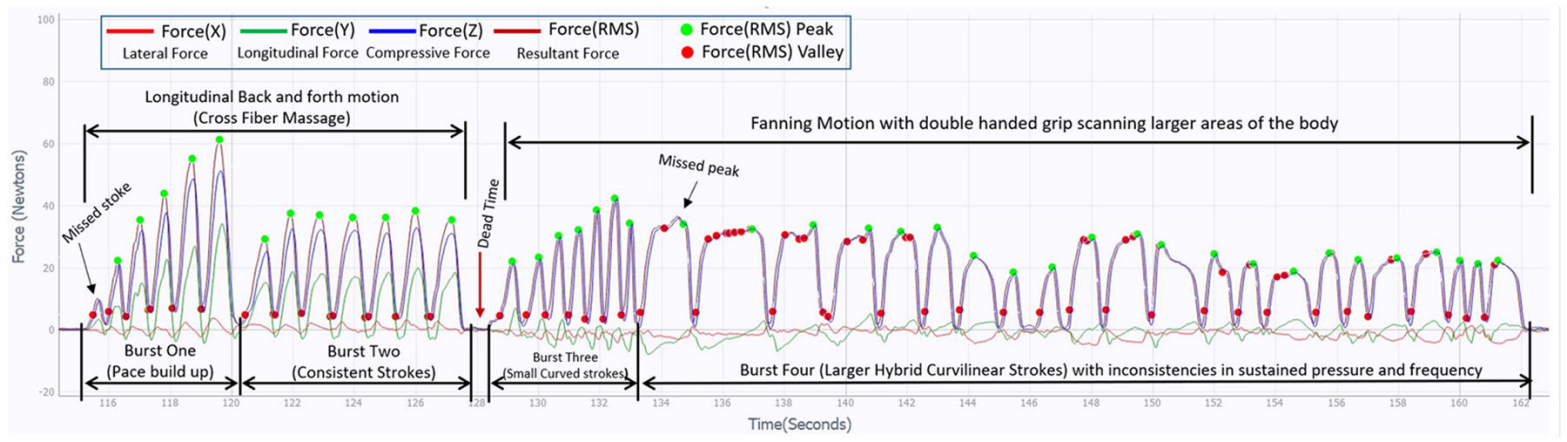
Graphical 3D force waveforms from Sub-session two of Fig. 10 capturing linear (longitudinal cross fiber massage) and hybrid curvilinear (fanning motion) force-motion patterns which depicts discreet combination of planar and compressive force component waveforms. The treatment burst patterns (train of similar paced force-motion cycles) progressions illustrate the nature of the treatment sub-session. The RMS force peaks and valleys shows the accuracy of the decision tree-based stroke count algorithm, as every force peaks (green dot) corresponds to a single force-motion cycle.

The decision tree-based stroke count algorithm has been validated with manual counts per visual recordings to identify false positives (missed peak) and missed strokes over stipulated skin contact time intervals. The computation of stroke frequency and bursts occur at the pause state after every sub-session as a part of post processing. The red dots in Fig. 11 depict the $F_{\text{RMS}}$ valley, while green dots are the peaks per stroke (force-motion cycle). Burst four in Fig. 11 represents a slow-paced curvilinear fanning motion. The red dots at the hill of second, third, fourth and successive strokes of burst four, indicates several redundant peak-valley pairs (due to hand vibration or soft tissue irregularity), which are successfully discarded to detect accurate stroke count and max peak per stroke in green dots. The stroke count algorithm proved to be 99% accurate when the device is used on inanimate objects, smooth tissue surfaces or rough tissue surfaces with slower rate as shown in Fig. 11. However, the accuracy level decreases to 90%, when the device is applied in varying orientations and directions over regions with uneven, curved, or non-uniform contours of human body (e.g., posterior thigh or calf muscle). Additional graphical observations also revealed that the accuracy of the stroke count algorithm varies with the rate of change of application i.e., the change in direction of stroke motions, change in contour of the treatment surface, or treatment pace. The average stroke frequency of every STM burst can be calculated to improve stroke count accuracy. Identification of a treatment burst can be computationally challenging for non-deterministic motions, as the user maneuvers and adapts to different force-motion portfolios at varying paces based on the instrumented palpation of the soft tissue region and treatment goal. Fig. 11 expands waveform of sub-session two from Fig. 10. It illustrates discreet combinations of different force-motion patterns with varying frequencies and directionalities of the planar force component progressions. Visual observations of bursts one and two from Fig. 11 indicates a cross fiber massage technique (i.e., linear back and forth motion parallel to the soft tissue fiber alignment) as the amplitude of the longitudinal force component along the y axis (in green) comprises a major part of the resultant force. While burst four show approximately consistent deflection from valley to peak along the y axis (longitudinal direction in green) for corresponding valley to valley forces along the z axis (compressive force component in blue), which illustrates that the device traversed a curvilinear path. This burst is called hybrid curvilinear motion as the frequency of the burst varies due to change of force amplitude as well as stroke length while performing a fanning motion with a single or double handed grip. Therefore, the dispersive handheld device enables identification of different treatment force-motion signatures, which can prove to be a clinical training tool or notation to reproduce and standardize dose-load regimens for replicable manual therapy.

### C. Clinical Case Study on Low Back Pain

To support the clinical usability of the developed dispersive QSTM device in quantifying treatment, a case study on a human subject with LBP was performed by an experienced manual therapist (>25 yrs experience) under prior approval of the Institutional Review Board at Indiana University. The subject suffered low back pain (>1 yr) from Lumbosacral grade-1 spondylolisthesis at L4-L5 segmental level with intersegmental disc degeneration evidenced by supporting radiographs. Four sessions were provided at 10 mins/session with 3-day intervals for 2 weeks using both the localized handheld QSTM device [25] and the dispersive handheld QSTM device, previously elaborated in this paper, for treating the LBP condition, based on a standard IASTM protocol (GRASTON technique) [8], [37]. The subject was not on any prescribed pain medications during the study. Functional and biological outcomes (trunk flexibility, soft tissue quality, static pain pressure threshold, SPPT) were measured pre- and post-treatment for all sessions using standardized clinical procedures including the modified-Schober’s test [38], MyotonPro [39], [40], and handheld algometer [41], [42], respectively. During SPPT testing, the subject was asked to indicate changes in pressure application from “comfortable to uncomfortable” by stating “now.” SPPT is inversely related to pain sensitivity. The average device to skin contact times were recorded to be 80.16% of total treatment time for combined use of both devices per session. The subject received a cold pack and instructions in gentle stretching exercises between sessions to reduce any potential soreness due to QSTM treatment.

**Fig. 12.**
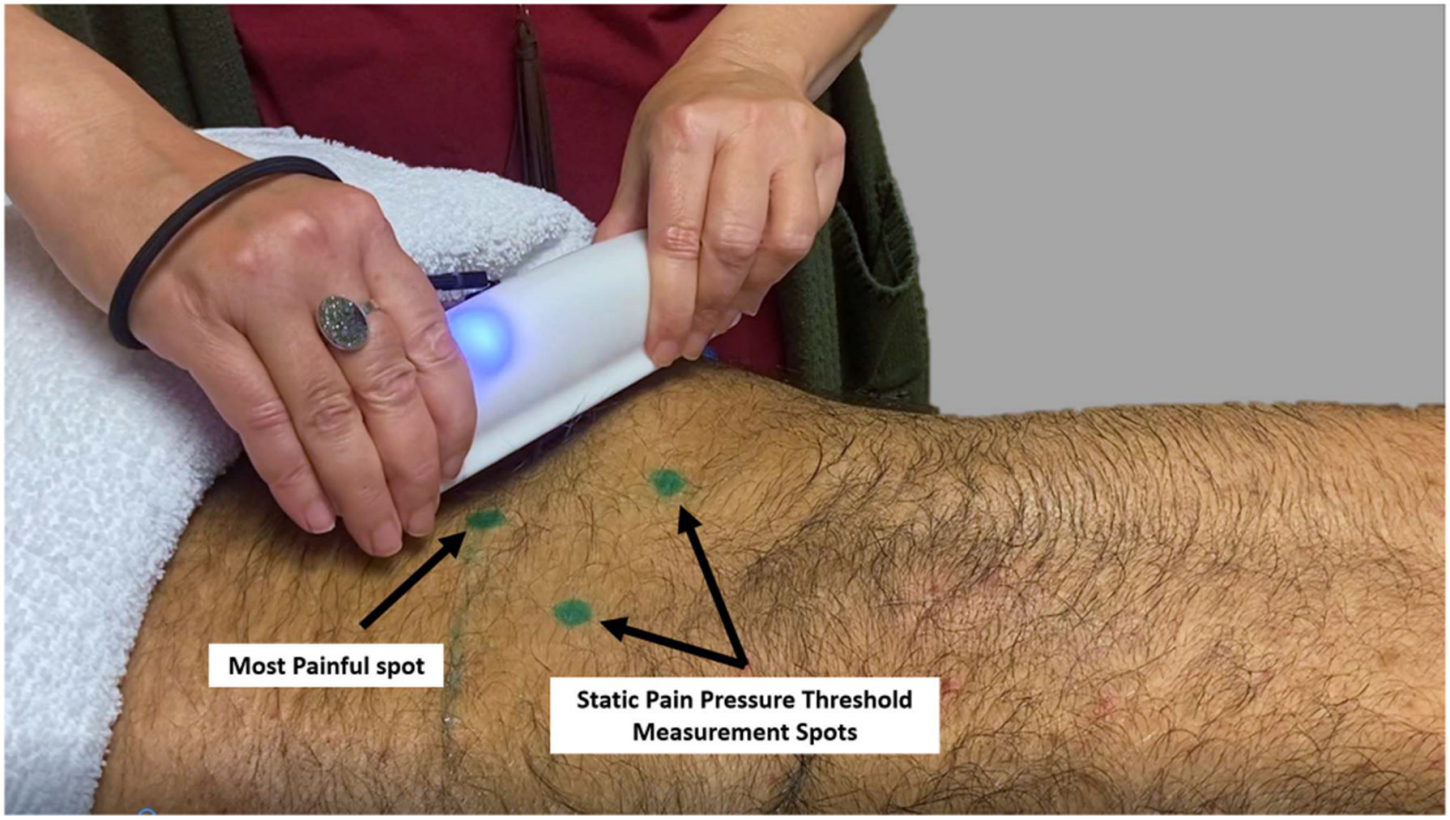
Clinical treatment of Subject with Grade-I spondylolisthesis with dispersive QSTM device. The green spots mark the area of the pain, where static pain pressure thresholds were taken pre-and post-treatment, bilaterally 3 cm lateral to L4 spinous process and the most painful spot.

The time taken by the device system from bootup to treatment ready state for the user to start STM application is approximately one minute, with an additional minute for adding post treatment remarks and bookmarking (2 minutes total). This time is reasonable with respect to clinical feasibility and information gained by using the dispersive QSTM device system. Documented QSTM treatment charts demonstrated force-motion patterns (linear types- Strumming and Scanning, Curvilinear types- Fanning and Sweeping) observed for a variety of treatment bursts of different stroke time-lengths and paces constituting a treatment session. The treatment force charts revealed initial pace building strokes during scanning the tissue followed by consistent force delivery for myofascial release. Average device to skin contact times were attributed to 47.22% for the localized device and 33.10% for dispersive device. Comparatively, the average STM dose regimen (average of resultant force peaks) was 2.4 times (137.5%) higher for dispersive device as compared to the localized one, whereas the force motion for the dispersive device were 41% slower with longer stroke lengths and a 20.6% steeper inclination to skin surface as compared to the localized device. Intra-session treatment report comparison showed 135% higher targeted force delivery on the last session as compared to the first. Improvements in soft tissue characteristics from first to last session were realized from the MyotonPro (9.9% less tissue stiffness, 3.4% less creep, 5.4% increased relaxation). The SPPT increased significantly across sessions (from first to last) representing a 73.58% increase in pressure tolerance i.e., lowered pain sensitivity at the most painful site (Fig. 12), after the last session. Eventually, steady improvements on self-reported pain levels reached an average 0/10, and 2/10 worse pain level after the fourth treatment, down from an average 7/10 and 9/10 worst pain levels before first treatment session. The overall positive results and gradual pain level improvements documented in the case study establishes the clinical feasibility of QSTM medical device system for research and clinical use for reproducible manually therapy. However, clinical trials are needed to determine the fidelity and efficacy of this novel technology, and study dose-load response in a variety of NMSK treatments and interventions.

## V. Discussion

The handheld force-motion tracking medical device along with its user-friendly operating software Q-Ware© described in this research successfully characterizes clinical manual therapy treatments in the form of Quantifiable Soft Tissue Manipulation (QSTM). The corresponding visual graphics on Q-Ware© identifies a variety of visually distinguishable force-motion patterns applied in manual therapy treatment, for pain assessment and treatment replication. Both the device firmware and Q-Ware© on PC was found to be robust and reliable, as the variable frame rate of GVI in Q-Ware© during real-time data-visualization optimizes response time and data storage. GVI in Q-Ware© offers the user to set a “Target Force Trendline”, during treatment, with which the user can apply targeted peak force per stroke cycle during application while visually monitoring the PC screen. The 3D force-motion waveforms recorded during treatment sessions of LBP, unveil identical signatures of linear or curvilinear stroke patterns applied in different directions by the clinician. The clinical assessment of the case study performed on the human subject with low back pain showed promising results with gradual progression in flexibility, soft tissue quality, and pressure pain tolerance of the subject leading to self-reported pain reduction. Thus, QSTM technology not only offers objective metrics to quantify manual therapy but also presents means to advance state-of-the-art practice and a common language for manual therapy prescription. Continued development is required to improve device precision especially in the areas of (a) adaptive self-calibration to optimize force baseline drifts due to prolonged treatments (more than 60 mins); (b) absolute pose estimation and orientation tracking in non-deterministic motions by introducing magnetometer in sensor fusion and eliminating hard and soft iron offsets; (c) improving treatment burst identification for hybrid burst patterns; and (d) estimating the elevation of device with respect to the changing tissue contour during dynamic force-motion applications. Future work will consider the reproducibility and reliability of the device system as used on humans and will compare QSTM with existing IASTM approaches to assess the effectiveness of this novel technology. Additionally, clinical trials with QSTM are needed to establish STM dose optimization across the varied human Body Mass Index (BMI) spectrum. Findings from the upcoming clinical trials could enable this device technology to be mounted on Robotic arms for remote telerehabilitation using force-motion pattern signatures in space stations or military base camps where intervention by a manual therapist aren’t possible.

## VI. Conclusion

The novel handheld mechatronic smart medical device illustrated in this paper is a one of its kind, which quantitates manual therapy using objective treatment parameters as a key to precision rehabilitation. It offers both targeted STM dose-load delivery with software guided feedback as well as adaptable maneuverability by the practitioner, required for individualized care of NMSK conditions. The validation results show accurate quantitated force measurements and angular orientation estimation of the device with minimal error, post proper calibration. This quantifiable IASTM medical device system proved to be practical for clinical use without significantly increasing the treatment time compared to hands-alone manual therapy. The fidelity and precision of the device enables accurate detection of stroke frequencies up-to 5 Hz. The force measurement accuracies worked best within the force measurement range of 0.2N to 325 N. Hence this medical device is suited to quantify STM treatments for a varied spectrum of patients with high to low pain tolerances. Findings from the clinical case study demonstrate usability of the system and show positive outcomes in an individual with low back pain. This is evidenced by reduced self-reported pain levels in conjunction with elevated magnitude of dose-loads tolerated by the human subject at the last treatment session as compared to the first. A broad spectrum of clinical trials with this smart medical device technology are necessary to substantiate the scientific rigor of QSTM prescriptions augmenting biological and functional outcomes of precision rehabilitation. The dispersive device in combination with the localized device are needed for clinical practice to advance manual therapy with software guided metrics. Future directions for QSTM are aimed at complementing dynamic pain algometry with enhanced soft tissue diagnostics for expedited recovery from NMSK disorders.
